# Atypical Early Onset Alzheimer’s Disease in a Young Female: A Case Report

**DOI:** 10.7759/cureus.78082

**Published:** 2025-01-27

**Authors:** Amina Namrouti, Philip Desamour, Alan Marquez, Carina Lorenzen, Odaimy Cervantes, Tate B Hodges, Giovanna Alvarez, Seema Chandra

**Affiliations:** 1 Medicine, Herbert Wertheim College of Medicine, Florida International University, Miami, USA; 2 Health Sciences, Herbert Wertheim College of Medicine, Florida International University, Miami, USA; 3 Family Medicine, Baptist Health South Florida, Miami, USA; 4 Radiology, Baptist Health South Florida, Miami, USA; 5 Internal Medicine, Baptist Hospital of Miami, Miami, USA

**Keywords:** acute encephalitis, brain fdg pet, early onset alzheimer's disease, mri brain scan, rapidly progressive dementia

## Abstract

Early-onset Alzheimer's disease (AD) is a neurodegenerative disorder characterized by the development of amyloid plaques and neurofibrillary tangles at an earlier age. It affects multiple cognitive domains, including memory, executive function, and motor abilities. Here, we present a case of atypical early-onset AD.

A 33-year-old woman with no significant medical history experienced a two-year decline in cognitive function, resulting in job loss and incidents such as cooking-related fires. Neurological examination revealed impaired attention, myoclonus, hyperreflexia, and a dystaxic gait. Imaging and tests showed minor abnormalities, with normal cerebrospinal fluid (CSF), genetic, autoimmune, and metabolic workups. Brain magnetic resonance imaging (MRI) and positron emission tomography (PET) fluorodeoxyglucose (FDG) scans indicated cortical atrophy and parietal hypometabolism. The patient was referred to a memory center for further evaluation and potential treatment with lecanemab.

This case highlights the challenges in diagnosing early-onset neurodegenerative disorders, which can present atypically and mimic other conditions. The extensive diagnostic workup emphasizes the difficulty of diagnosing these disorders, particularly in the absence of specific biomarkers.

Early diagnosis of neurodegenerative disorders in young adults requires heightened clinical suspicion and a comprehensive diagnostic workup, including advanced brain imaging, such as MRI and PET scans, to ensure timely diagnosis and referral to specialized centers.

## Introduction

Alzheimer's disease (AD) is a neurodegenerative disorder characterized by amyloid plaque deposition and neurofibrillary tangles, typically beginning in the seventh or eighth decades of life [1]. However, about 3% of the 6 million Americans with AD develop symptoms before age 65, termed early-onset AD [1]. Early-onset AD significantly impacts multiple cognitive domains, including memory, executive function, and motor abilities, and presents more aggressively than late-onset AD [2]. Recent literature suggests an increasing prevalence of early-onset AD, highlighting the need for further research [2].

Early-onset dementia (EOD) can result from various causes, including AD, vascular dementia, frontotemporal dementia, and others like Huntington’s or Parkinson’s disease [2,3,4]. Reversible causes, such as autoimmune or infectious conditions, toxins, or metabolic disorders, are additional possible causes [2,3,4]. EOD often has a genetic basis, with autosomal dominant inheritance being common. The apolipoprotein E (APOE) e4 allele is a known risk factor for early-onset AD [5]. Compared to late-onset AD, EOD progresses faster and is associated with atypical symptoms, such as myoclonus, seizures, or gait disturbances, affecting individuals in their prime working and social years [2,3,5].

Diagnosing EOD is challenging due to its rarity and atypical presentation. A comprehensive workup includes clinical evaluation, cognitive tests, imaging, lumbar puncture, and genetic testing. Advanced imaging, such as magnetic resonance imaging (MRI) and fluorodeoxyglucose positron emission tomography (FDG-PET) scans, typically reveal widespread cortical atrophy, especially in the parietal cortex. Treatment options are limited to symptom management through medications, therapy, and support services. Life expectancy is shorter in EOD due to its aggressive nature [3,4,5].

In this study, we report a case of a 33-year-old woman with early-onset AD who presented to our hospital with complex neurological deterioration, adding to the growing literature on this rare but important condition. 

## Case presentation

A 33-year-old woman with no significant medical history presented with a two-year-long history of progressive cognitive decline. Her husband reported that, after moving from Cuba four years ago, she lost her housekeeping job due to forgetfulness and difficulty completing tasks. Over the past year, she lost four additional jobs due to an inability to follow simple instructions. She had unintentionally caused two house fires by leaving the stove on and had other incidents of confusion, such as greeting the same person multiple times and not realizing she was menstruating. She also experienced ataxia, abnormal movements, and a weight loss of approximately 30 pounds. Despite consultations with two neurologists, no definitive diagnosis was reached. 

She had no history of infections, recent medication changes, toxic exposures, or substance abuse. Notably, her father had died of leptospirosis and exhibited cognitive decline in his 40s, though the details were unclear. 

On admission, she was alert but struggled with recall and following two-step commands. Neurological examination revealed intermittent involuntary movements, asterixis, bilateral myoclonus, global hyperreflexia, and a dystaxic gait. Coordination tests, such as dysdiadochokinesia and finger-to-nose testing, were abnormal, but nystagmus, fasciculations, and significant cranial nerve abnormalities were absent. Differential diagnoses included paraneoplastic or autoimmune encephalitis, prion disease, Huntington’s disease, subacute sclerosing panencephalitis, early-onset AD, and various infectious or metabolic disorders.

Genetic testing for Huntington’s disease was negative, and a broad autoantibody and paraneoplastic panel for autoimmune and paraneoplastic causes was unrevealing. Chest X-ray and computed tomography (CT) of the abdomen and pelvis were also unremarkable. The patient had no evidence of infections such as human immunodeficiency virus (HIV), human T-lymphotropic virus types 1 and 2 (HTLV I/II), syphilis, or John Cunningham virus (JC virus). Metabolic workup showed normal levels of vitamin B12, folate, thiamine, and glucose, though elevated copper (209) and mildly low vitamin E (5.3) were noted. Additional tests, including serum protein electrophoresis and methylenetetrahydrofolate reductase (MTHFR) DNA analysis, were unremarkable. Further serological testing showed immunity against rubeola immunoglobulin (IgG), reducing the likelihood of subacute sclerosing panencephalitis.

**Figure 1:**
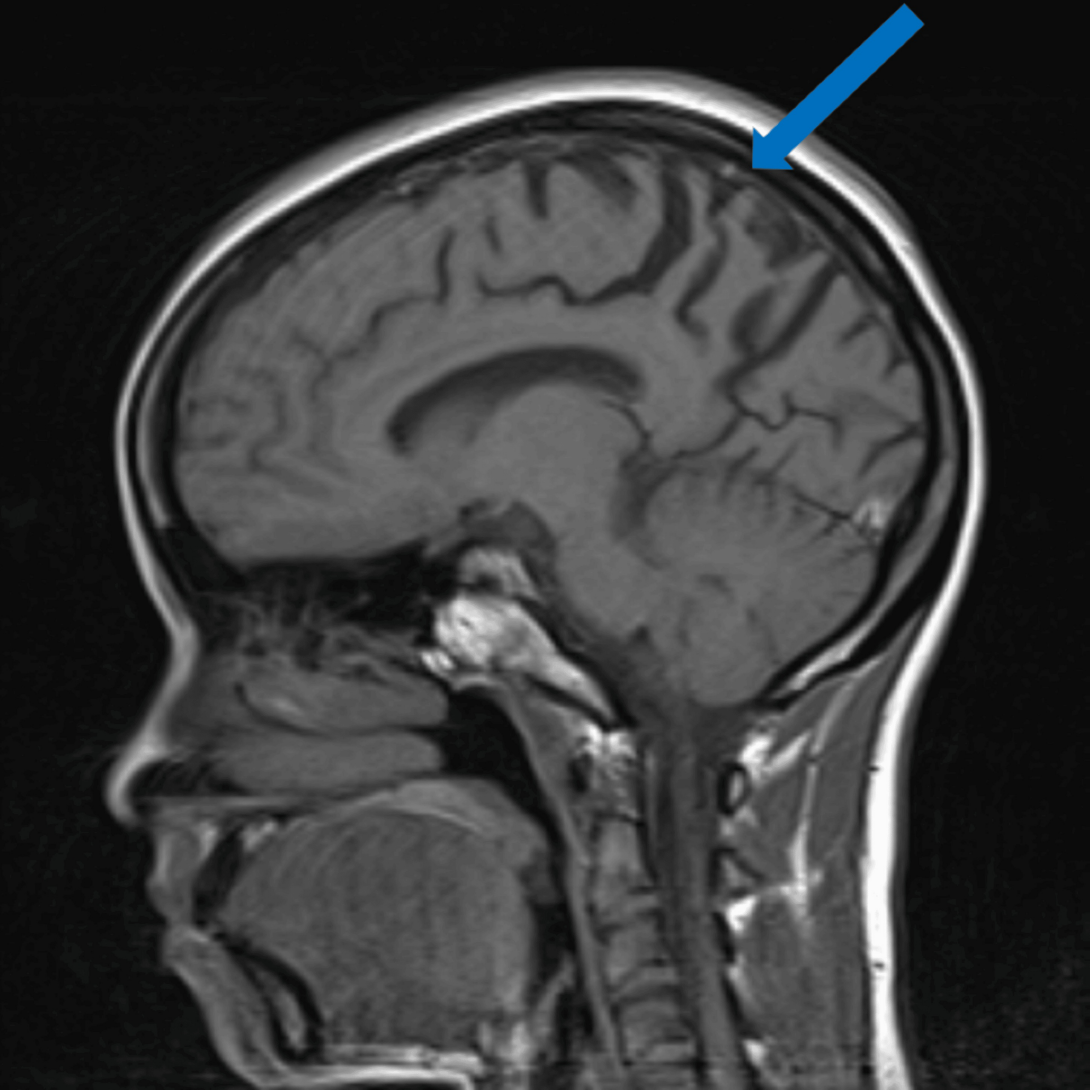
Sagittal MRI brain T1 imaging illustrating bilateral parietal lobe cortical atrophy. The blue arrow points to the region of reduced cerebral volume and illustrates the widening of cerebral sulci in the parietal lobe, consistent with cortical atrophy. MRI, magnetic resonance imaging

**Figure 2:**
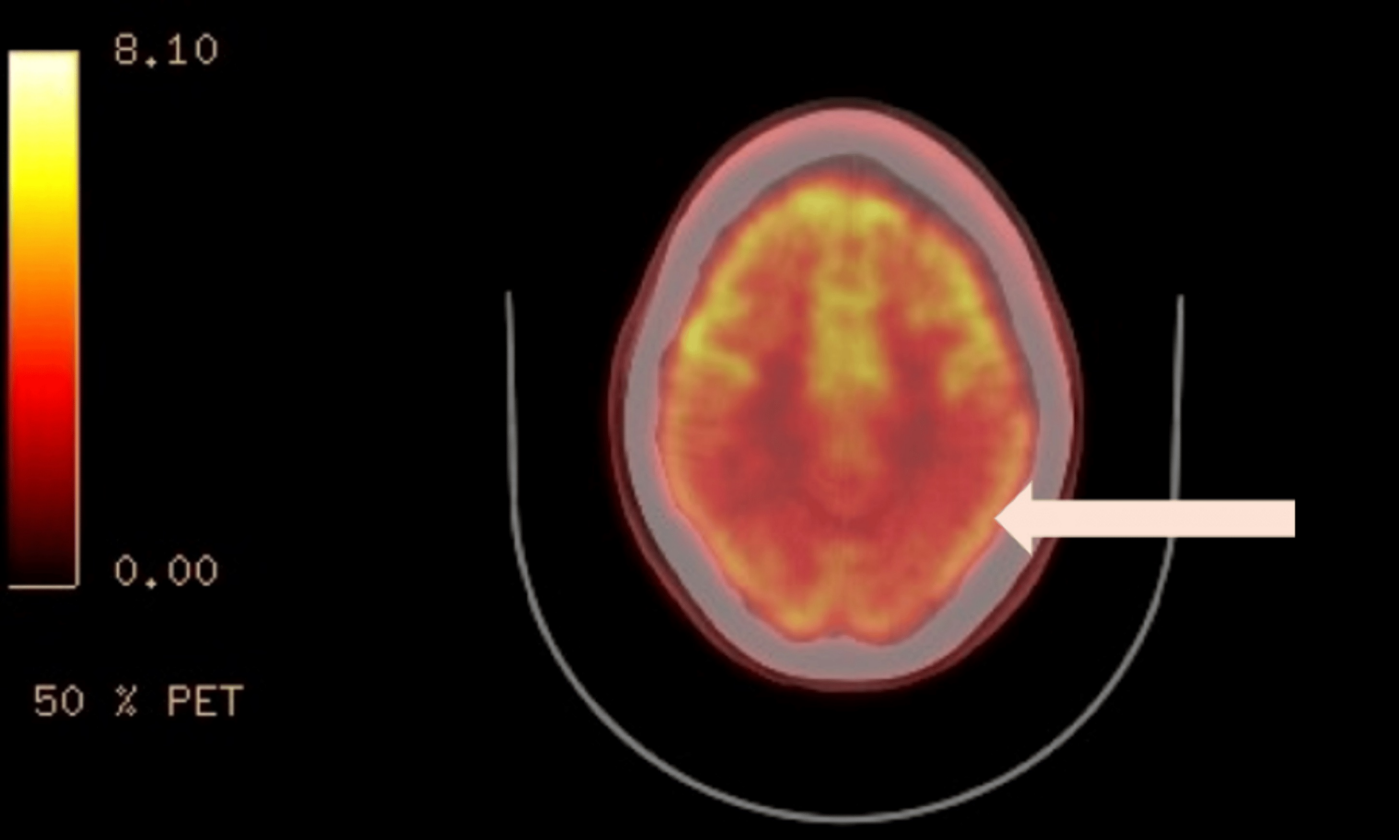
Axial fused 18F-fluorodeoxyglucose positron emission tomography/computed tomography (18F-FDG PET/CT) demonstrates moderate-significant decreased FDG uptake in the bilateral parietal lobes. The white arrow points to bilateral parietal lobes with FDG hypometabolism, signifying neuronal dysfunction and potential neurodegenerative changes.

A lumbar puncture revealed normal cerebrospinal fluid (CSF) analysis, with no signs of infection, inflammation, or malignancy. Both 14-3-3 protein and real-time quaking-induced conversion (RT-QuIC) were negative, indicating a low likelihood of prion disease. However, elevated 14-3-3 gamma protein (3805), with normal tau protein levels (517), was noted. An inpatient MRI showed cortical atrophy, primarily in the parietal regions, without caudate head involvement. FDG PET scan demonstrated hypometabolism in the bilateral parietal lobes (Figures 1-2). These findings, along with the clinical presentation, led to a diagnosis of atypical early-onset AD. Further evaluation revealed elevated cortisol levels and an enlarged pituitary gland, though pituitary function tests were normal. Elevated liver function tests were also noted but trended down during hospitalization, and extensive workup for these abnormalities was unrevealing.

The final diagnosis was atypical early-onset AD. The patient was advised to follow up with an outpatient memory center for further evaluation and consideration of treatment options, such as lecanemab.

## Discussion

This case presents a complex diagnostic challenge involving a young female patient with progressive cognitive decline and neurological symptoms. The initial presentation of deteriorating cognitive function in multiple domains, difficulty with simple tasks, compounded by neurological symptoms such as ataxia and involuntary movements, raised a wide range of differential diagnoses.

Initial considerations included prion disease, Huntington's disease, metabolic derangements, autoimmune encephalitis, and others due to overlapping clinical features. However, negative genetic testing for Huntington's, absence of autoantibodies indicative of autoimmune encephalitis or paraneoplastic syndrome, and a negative infectious and metabolic workup, along with normal CSF analysis - including unremarkable 14-3-3 and RT-QuIC results typically seen in prion disease - helped narrow the differential [6,7].

Neuroimaging, including MRI and PET FDG scan, revealed cortical atrophy and hypometabolism in the parietal and occipital regions, suggestive of neurodegenerative changes. These findings suggested a neurodegenerative disorder, with atypical early-onset AD becoming the most likely diagnosis. The persistence and worsening of symptoms over time, despite supportive care, highlight the progressive nature and severity of the syndrome.

The lack of specific biomarkers or diagnostic criteria for certain neurodegenerative disorders, such as early-onset AD, serves as a limitation, making this a diagnosis of exclusion. The patient notably had nonspecific positive proteins in the CSF (elevated 14-3-3 gamma), which have been associated with AD. 14-3-3 gamma has been linked to neurodegeneration, potentially influencing tau phosphorylation and leading to neurofibrillary tangles, a key process in AD pathology [6]. Though its diagnostic value is less specific than tau or beta-amyloid, the absence of a clear family medical history and discharge to an outside memory center leaves her prognosis and treatment plan unclear, limiting insight into long-term outcomes and therapeutic efficacy.

This case demonstrates the inherent challenges in diagnosing early-onset neurodegenerative disorders, which may present with atypical features and mimic other conditions, such as prion disease, Huntington’s disease, or autoimmune encephalitis. The extensive diagnostic workup highlights the difficulty in reaching a definitive diagnosis, especially in the absence of specific biomarkers, but demonstrates how the utilization of advanced brain imaging techniques, such as MRI and PET scan, and CSF protein analysis aids in reaching a diagnosis.

This case offers valuable insight, highlighting the importance of recognizing atypical presentations in young adults. Increased awareness can improve diagnostic accuracy and reduce treatment delays. Early referral to specialized memory centers is essential for timely diagnosis, slowing progression, and accessing emerging therapies.

Further research is needed to understand the progression of atypical neurodegenerative disorders, refine diagnostic biomarkers, and improve management strategies, ultimately benefiting patient outcomes and quality of life. 

## Conclusions

This case highlights the challenge of diagnosing early-onset neurodegenerative disorders, especially with atypical presentations in young adults. It underscores the importance of a broad differential diagnosis, utilization of MRI and PET scans, and CSF analysis for t-tau and 14-3-3 gamma, for timely diagnosis, early referral to specialized centers, and a multidisciplinary approach. Continued research and development of sensitive diagnostic tools are crucial for improving outcomes and advancing treatment options.
